# Maternal bis-glycinate bound zinc supplementation alters sow performance and milk metabolomic-lipidomic profiles and mitigates piglet diarrhea

**DOI:** 10.1016/j.vas.2026.100697

**Published:** 2026-05-12

**Authors:** Jakavat Ruampatana, Sarn Settachaimongkon, Nathathai Soodsaward, Banthita Chuaydamrong, Ponlatorn Rukklang, Suphacha Boonyasantisuk, Suphasorn Ratchatakajornkit, Unchean Yamsrikaew, Kunaporn Homyog, Rawiwan Kuabphimai, Lampetch Vimuktalop, Junpen Suwimonteerabutr, Morakot Nuntapaitoon

**Affiliations:** a Department of Obstetrics, Gynaecology and Reproduction, Faculty of Veterinary Science, Chulalongkorn University, Bangkok 10330, Thailand; b Multi-Omics for Functional Products in Food, Cosmetics and Animals Research Unit, Chulalongkorn University, Bangkok 10330, Thailand; c Department of Food Technology, Faculty of Science, Chulalongkorn University, Bangkok 10330, Thailand; d Omics Sciences and Bioinformatics Center, Faculty of Science, Chulalongkorn University, Bangkok 10330, Thailand; e Center of Veterinary Diagnosis, Faculty of Veterinary Science, Mahidol University, Nakhon Pathom, 73170, Thailand; f Scientific and Technological Research Equipment Center (STREC), Chulalongkorn University, Bangkok 10330, Thailand

**Keywords:** Bis-glycinate bound zinc, Diarrhea, Lipidomics, Metabolomics, Milk, Sow

## Abstract

Dietary zinc supplementation to piglets reduces diarrhea and improves growth, yet the effects of maternal zinc supplementation on milk and subsequent piglet performance remain unclear. This study evaluated the impact of maternal zinc supplementation on sow, piglet, and lactation performances. Thirty-six crossbred sows were randomized to CON (standard diet; *n* = 18) or TRT (standard diet supplemented with 1 g/sow/day of bis-glycinate bound zinc; *n* = 18) from day 85 of gestation to day 21 of lactation. Sow and piglet performance were recorded. Blood was collected at farrowing to determine serum malondialdehyde (MDA). Colostrum and milk (on days 3 and 10 of lactation) were analyzed for metabolomics and lipidomic profiles. No difference was observed in total number of piglets born, but the percentage of mummified fetuses was higher in TRT than in CON sows (*P* = 0.030). The TRT sows exhibited greater relative backfat thickness loss during lactation (*P* = 0.024) and higher serum MDA at farrowing (*P* = 0.033). Piglets nursed by TRT sows tended toward higher colostrum intake (*P* = 0.056), higher weight on day 21 (*P* = 0.116), and lower diarrhea incidence on days 2, 4, 5, 15, and 21 (*P* < 0.05; for all). In mature milk, TRT sows had higher contents of fat (*P* = 0.023) and total solids (*P* = 0.014), while lower lactose (*P* = 0.028). Metabolomic and lipidomic analyses of mature milk revealed significantly higher concentrations of *O*-acetylcarnitine, *O*-acetylcholine, *sn*‑glycero-3-phosphocholine, creatine, uridine monophosphate, citrate, dimethylamine, arachidic acid, linolelaidic acid, eicosadienoic acid, and arachidonic acid in TRT. These compounds indicate shifts in energy and lipid metabolism and were positively associated with piglet growth. In conclusion, maternal bis-glycinate bound zinc supplementation was associated with changes in backfat thickness mobilization during lactation, and alterations in milk composition, thereby improving piglet performance.

## Highlights

• Maternal zinc supplementation was associated with greater backfat thickness loss during lactation.
• Maternal zinc supplementation resulted in higher fat and total solids, while lower in lactose in mature milk.
• Maternal zinc supplementation modulated both metabolomic and lipidomic profiles in colostrum and milk.
• Piglets nursed by zinc-supplemented sows tended to have higher body weight on day 21.
• Piglets nursed by zinc-supplemented sows exhibited lower diarrhea incidence.

## Graphical abstract

**Figure:**
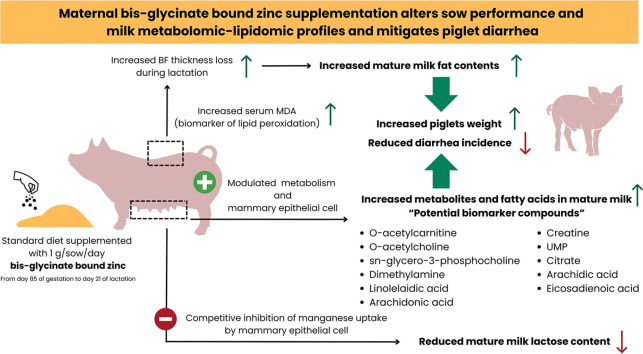

## 1 Introduction

Pre-weaning mortality (PWM) in piglets remains a major challenge in the swine industry, as genetic selection for increased litter size has been associated with a rise in PWM (Nuntapaitoon & Tummaruk, 2015, 2018; Rahman et al., 2023). Both infectious and non-infectious factors contribute to PWM (Christensen & Svensmark, 1997; Muns et al., 2016), with neonatal diarrhea recognized as one of the common causes of mortality in piglets during the pre-weaning period (Christensen & Svensmark, 1997; Svensmark et al., 1989). Previous studies have shown that direct supplementation with zinc in piglets reduces diarrhea incidence through antibacterial effects and by improving intestinal barrier function and microbial balance (Tang et al., 2024), while maternal zinc supplementation has been associated with lower piglet mortality (Hammers et al., 2024; Holen et al., 2020). Accordingly, zinc has been considered a functional nutritional strategy to mitigate PWM.

Zinc is an essential trace mineral that supports various metabolic functions in animals (Alimohamady et al., 2019; Duffy et al., 2023). As a functional nutritional additive, zinc supplementation has been shown to improve average daily gain and feed intake, and reduce diarrhea incidence in weanling piglets (Huang et al., 2024; Tang et al., 2024). Barszcz et al. (2019) demonstrated that different zinc sources (organic or inorganic) exert distinct effects on piglet growth and nutrient digestibility. Zinc sulfate, an inorganic form, can react with metal ions to generate free radicals, leading to the degradation of vitamins, fatty acids (FA), and other nutrients (Zhang & Guo, 2007). Additionally, it may form complexes with phytase, reducing zinc absorption in the gut (Schlegel & Windisch, 2006). In contrast, zinc chelated with glycine, an organic form bound to amino acids, offers improved absorption and bioavailability in the small intestine, which enhances piglet performance (Barszcz et al., 2019; Byrne et al., 2021). However, the potential benefits of bis-glycinate bound zinc supplementation in sows during late gestation and lactation, particularly its effect on colostrum and milk nutritional composition and subsequent piglet performance, have not yet been fully elucidated. Previous studies have shown that supplementation with various functional nutritional additives during late gestation and lactation modulates colostrum and milk nutritional composition, thereby reducing piglet diarrhea (Nuntapaitoon et al., 2025; Ruampatana et al., 2024, 2025a; Wongsamart et al., 2025). Therefore, we hypothesized that bis-glycinate bound zinc supplementation during this period exerts similar beneficial effects on colostrum and milk nutritional composition, ultimately improving piglet health and growth. Accordingly, this study aimed to evaluate the effects of maternal bis-glycinate bound zinc supplementation during late gestation and lactation on sow and piglet performance, colostrum and milk nutritional composition, and piglet diarrhea incidence.

## 2 Materials and methods

### 2.1 Experimental design

The use of animals in this experiment was approved by the Institutional Animal Care and Use Committee at Chulalongkorn University (Approval number: 2331017).

The experiment was conducted on a commercial swine farm in Ratchaburi province, Thailand. A total of 36 Landrace × Yorkshire crossbred sows (average parity 2.9 ± 1.9; ranging from 1 to 6) were involved. On day 85 of gestation, the sows were divided into two groups: CON group (*n* = 18; parity 1 = 7, parity 2–4 = 7; parity 5–6 = 4), which was fed a standard diet, and TRT group (*n* = 18; parity 1 = 7, parity 2–4 = 6; parity 5–6 = 5), which was fed a standard diet supplemented with 1 g/sow/day of bis-glycinate bound zinc (Plexomin Zn®; Phytobiotics North America, Cary, NC, USA) by top-dressing from day 85 of gestation until day 21 of lactation (52.3 ± 2.1 days). According to manufacturer specifications, Plexomin® Zn contains 26% zinc derived from organic sources. Previous studies have demonstrated that maternal supplementation with organic zinc source can improve pre-weaning piglet performance and modulate gut microbiota.Dietary inclusion of an additional 100 ppm zinc from zinc amino acid complexes (∼270 mg zinc/day based on an average feed intake of 2.7 kg/day) improves pre-weaning piglet performance (Payne et al., 2006), whereas supplementation with 1 g/sow/day of bis-glycinate bound zinc mdualted gut microbiota in both sows and piglets (Somboonna et al., 2026). In this study, a comparable supplementation level was applied, providing approximately 260 mg zinc/day through top-dressing 1 g/sow/day of bis-glycinate-bound zinc.

### 2.2 Housing, diet, and general management

During gestation, gilts and sows were housed individually in stalls within a gestation house with an evaporative cooling system and fed with 3 kg/day of standard gestation diet from the day of insemination until day 108 of gestation. Table 1 presents the ingredients of the standard gestation and lactation diets. The standard gestation diet (as-fed basis) contained 11.8% crude protein, 5.7% crude fat, 4.6% crude fiber, 11.3% ash, and 9.7% moisture. On day 109 of gestation, gilts and sows were moved to the farrowing house with an evaporative cooling system and kept in individual farrowing crates equipped with a piglet creep area until weaning. During this period, sows were fed a standard lactation diet (3 kg/day). After farrowing, the feed allowance was increased by 0.5 kg/day until it reached 6.0 kg/day. The standard lactation diet, which contained (as-fed basis) of 16.2% crude protein, 8.4% crude fat, 4.67% crude fiber, 7.5% ash, and 10.0% moisture.

**Table 1:** Dietary ingredients and nutrient composition of the experimental diets (as-fed basis).

Item	Content	
	Gestation diet	Lactation diet
Ingredients (g/kg)		
Corn	323	472
Broken rice	323	149
Cassava	199	74.6
Soybean meal	104	244
Poultry meal	-	22.4
Soybean oil	7.46	32.3
Dicalcium phosphate	12.4	14.9
Limestone	17.4	14.9
Salt	3.98	3.98
Lysine	1.49	2.74
Methionine	0.52	0.25
Threonine	1.00	1.00
Mycotoxin binder	1.00	1.00
Premix^1^	1.00	1.00

1 The premix provided the following nutrients per kilogram: vitamin A (16,000 IU), vitamin D₃ (3250 IU), vitamin E (60 IU), vitamin K_3_(2 mg), vitamin B_1_ (4 mg), vitamin B_2_ (8 mg), vitamin B_3_ (6 mg), vitamin B_12_ (0.03 mg), vitamin C (0.15 mg), pantothenic acid (16 mg), niacin (30 mg), folic acid (2 mg), biotin (0.35 mg), choline (450 mg), manganese (60 mg), iron (120 mg), zinc (125 mg), cobalt (2 mg), copper (12 mg), iodine (0.25 mg), selenium (0.2 mg), and magnesium (65 mg).

Farrowing assistance was provided when dystocia was suspected, defined as active straining without piglet expulsion for 45 min or an inter-piglet interval > 45 min. After the delivery of the eighth piglet, sows were administered 10 IU of oxytocin intramuscularly (VetOne®, MWI Veterinary Supply Co., Ltd., Boise, ID, USA) to facilitate farrowing. Water was provided ad libitum to sows and piglets. On day 1 of age, piglets were tail-docked, teeth clipped, and administered 1 mL of iron intramuscularly (10% IRON®, Big Chemical CO., Ltd., Nakhon Pathom, Thailand). On day 3 of age, piglets received an oral anticoccidial (Baycox®, OLIC Ltd., Ayutthaya, Thailand). Litters were standardized to 14 piglets/sow.

### 2.3 Data collection for sow and piglet and determination of colostrum and milk yield

The sow data included sow identity, parity, insemination date, farrowing date, backfat thickness (BFT), total number of piglets born (TB), number of piglets born alive (BA), percentage of stillborn piglets (SB), and percentage of mummified fetuses (MM). The BFT was measured on days 85 and 109 of gestation and on day 21 of lactation, using A-mode ultrasonography (Renco Lean-Meater®, MN, USA) at the last rib, approximately 6.5 cm from the midline. The relative BFT loss during lactation was calculated as the percentage reduction between day 109 of gestation and day 21 of lactation.

On the day of farrowing, teat functionality was assessed based on colostrum ejection, following the method adapted from Balzani et al. (2016). A functional teat was defined by successful colostrum ejection after a gentle massage or a stripping action applied with the thumb and forefinger from the mid-mammary gland toward the teat end. In contrast, a nonfunctional teat was characterized by the absence of colostrum ejection following the same stimulation procedure. The total number of functional teats per sow was calculated as the sum of teats classified as functional.

For the piglet data, the time of farrowing and individual body weight were measured at birth and 24 h after birth, using an electronic digital balance (B6S Weighing Indicator, ZEPPER Instrument Co., Ltd., Nonthaburi, Thailand). The individual piglet data were then used to estimate colostrum intake (CI) using the equation described by Theil et al. (2014):

$$\mathrm{CI}\left(g\right)=-106+2.26\;\mathrm{WG}+200BW_{B}+0.111D+1414\frac{\mathrm{WG}}{D}+0.0182\;\mathrm{WG}/BW_{B}$$

Where WG = weight gain (g), BW_B_ = birth weight (kg), and D = duration of colostrum suckling (mins). Colostrum yield (CY) was estimated by summing the total CI of all piglets within each litter.

The litter size and litter weight were recorded on days 3, 10, and 21 of age. Average piglet body weight at each time point was calculated by dividing litter weight by litter size. These data were used to estimate stage-specific milk production using the equation described by Hansen et al. (2012). A mathematical model was applied to calculate milk yield (MY) during days 3–10 and days 10–21 of lactation.

$$MY\;\left(kg\right)=2.23+0.05\;\left(litter\;size-9.5\right)+0.23\;\times \;\left(dailu\;weight\;gain\;\left(\frac{kg}{day}\right)\right)-2.5$$

### 2.4 Incidence of diarrhea in piglets

Piglet fecal consistency was evaluated daily in each farrowing crate by trained observers. The fecal characteristics were scored using a four-point scale: (0 = solid, 1 = semi-solid, 2 = semi-liquid, and 3 = liquid). Piglets with scores ≥ 2 were considered diarrheic. The incidence of diarrhea was calculated according to the method previously described by Ruampatana et al. (2025a):

$$diarrhea\;incidence\;\left(\%\right)=\;\frac{number\;of\;piglets\;with\;diarrhea\;in\;each\;litter}{litter\;size}\;\times \;100$$

### 2.5 Blood collection

Blood samples (5 mL) were collected from sows within 1 h after the birth of the first piglet using a 21-gauge, 1-inch needle via the auricular vein. The site was disinfected with 75% alcohol before collection. After collection, blood samples were allowed to coagulate at room temperature, then centrifuged at 2000 × *g* for 15 min to separate the serum, which was stored at −20 °C until analysis.

### 2.6 Colostrum and milk collection

A total of 30 mL of colostrum was collected from all functional teats within 1 h after the birth of the first piglet. For milk collection, 0.2 mL of oxytocin (10 IU/mL; General Drugs House Co. Ltd., Bangkok, Thailand) was administered via the auricular vein. Milk samples (30 mL) were then collected from all functional mammary glands on days 3 (transient milk) and 10 of lactation (mature milk). The samples were filtered through sterile gauze, transferred into sterile bottles, and stored at −20 °C until analysis.

### 2.7 Determination of malondialdehyde concentration in serum

Serum malondialdehyde (MDA) concentrations in sows were quantified using a thiobarbituric acid reactive substances (TBARS) assay kit (Cell Biolabs, Inc., SD, USA) in accordance with the manufacturer’s instructions. Briefly, 100 µL of serum was mixed with an equal volume of sodium dodecyl sulfate lysis solution in a 1.5 mL microcentrifuge tube, then incubated at room temperature for 5 min. Thiobarbituric acid reagent (250 µL) was then added, and the reaction mixture was incubated at 95 °C for 60 min. After cooling on ice for 5 min, samples were centrifuged at 3000 × *g* for 15 min to separate the supernatants. The 300 µL supernatant was transferred to a clean tube, mixed with n-Butanol by vortexing for 2 min, and centrifuged at 10,000 × *g* for 5 min. Subsequently, 200 µL of the butanol layer and MDA standard were transferred to a 96-well microplate, and absorbance was read at 532 nm using an ELISA plate reader (Tecan Sunrise™, Männedorf, Switzerland).

### 2.8 Determination of major compositions and immunoglobulins in colostrum and milk

The major compositions of colostrum and milk, including fat, protein, lactose, casein, and total solids were analyzed using an infrared spectrometer method with the MilkoScan FT2 analyzer (Foss MilkoScan, Hillerød, Denmark). In addition, the concentrations of immunoglobulin G (IgG), IgA, and IgM in colostrum were quantified using Enzyme-linked immunosorbent assay kits (ELISA quantitation kit, Bethyl Laboratories Inc., Texas, USA), according to the procedure described by Nuntapaitoon et al. (2019a).

### 2.9 Non-volatile polar metabolite profiling by ^1^H-NMR technique in colostrum and milk

The non-volatile polar metabolites in sow colostrum and milk were profiled using a non-targeted proton nuclear magnetic resonance (^1^H-NMR ) metabolomics, following the previously published protocol by Settachaimongkon et al. (2023). Before analysis, colostrum and milk samples were adjusted to pH 6.0. Lipids and high-molecular-weight proteins were removed by dichloromethane extraction and ultracentrifugation (74,200 × g for 60 min at 4 °C), respectively. The resulting supernatant was filtered through a Pall Nanosep® centrifugal device with a 3 kDa cutoff (Pall Life Sciences, Ann Arbor, MI, USA). The milk serum was subsequently combined with a phosphate buffer (pH 6.0) at a 1:1 (v/v) ratio, containing 1 mM 3-(Trimethylsilyl) propionic-2,2,3,3-d_4_ acid sodium salt (Merck, Darmstadt, Germany) as an internal standard.

The ^1^H-NMR spectra were acquired using a 600 MHz NOESY-GPPR-1D-¹H—NMR spectrometer (Bruker, Rheinstetten, Germany) under conditions consistent with Settachaimongkon et al. (2023). The spectra data were subjected to correction, pre-processing, and quantification. Metabolite identification was performed using Chenomx NMR suite 8.2 library (Chenomx Inc., Edmonton, AB, Canada), the Livestock Metabolome Database (www.lmdb.ca), and published references (Luise et al., 2020; Picone et al., 2018; Ruampatana et al., 2024, 2025a; 2025b; Settachaimongkon et al., 2023). The sum of ^1^H-NMR signal intensities of each metabolite was expressed as log_10_ transformed [arbitrary unit] and subsequently used as variables for statistical analysis.

### 2.10 Fatty acid profiling by GC/MS analysis in colostrum and milk

Fatty acid compositions of colostrum and milk were analyzed as fatty acid methyl esters using gas chromatography–mass spectrometry (GC/MS-FAME). Analyses were conducted with an Agilent 7890A-5975C (Agilent Technologies, Santa Clara, CA, USA), following an established protocol by Settachaimongkon et al. (2023). In brief, colostrum and milk samples were subjected to hydrolysis and methylation using KOH, methanol, and sulfuric acid under heating conditions. The resulting fatty acid methyl esters (FAME) were extracted with hexane before analysis. The FA compositions of the FAME were determined by capillary gas chromatography (GC) utilizing *An sp-*2560 capillary column (Supelco, Bellefonte, PA, USA). The FA were identified based on comparison of retention times and mass spectra (*m/z*) with a commercial reference standard (Supelco 37 Component FAME mix, Sigma-Aldrich, Steinheim, Germany). The FA concentration was determined by automated peak integration, and results are expressed as log_10_ transformed [arbitrary unit] and subsequently used as variables for statistical analysis.

### 2.11 Statistical analyses

All statistical analyses were performed using SAS (version 9.4, Cary, NC, USA). Descriptive statistics for continuous variables (parity and litter size on days 3, 10, and 21 of age) were analyzed using the MEANS. The effects of maternal zinc supplementation on sow performance (BFT on days 85, 109 of gestation and day 21 of lactation, the relative BFT loss during lactation, number of functional teats, TB, BA, SB, MM, CY, MY between days 3–10 and 10–21 of lactation), serum MDA, and colostrum IgG, IgA, and IgM were analyzed using the general linear model (PROC GLM), with dietary supplement (CON and TRT) included as a fixed effect. For the litter performance (average piglet weight on days 3, 10, and 21 of age), the statistical model included dietary supplementation as a fixed effect and the litter size as a covariate. The incidence of piglet diarrhea on each day was also analyzed using PROC GLM. The effects of maternal zinc supplementation on individual piglet weight at birth, 24 h after birth, and CI were analyzed using the generalized linear mixed models (PROC MIXED) procedure, with dietary supplementation as a fixed effect and sow identity as a random effect. The major and biomolecular nutritional compositions (non-volatile metabolites and FA) of colostrum and milk were analyzed using PROC MIXED, with the model including dietary supplementation, lactation stage (colostrum, transitional milk, and mature milk), and their interaction as fixed effects, and sow identity as a random effect. For all statistical tests, a *P*-value < 0.05 was considered statistically significant.

^1^H-NMR -derived metabolomic and GC/MS-derived lipidomic data were analyzed and compared by means of multivariate statistics in MetaboAnalyst 6.0 (www.metaboanalyst.ca). Partial least squares discriminant analysis (PLS-DA) was employed to illustrate distinct separation patterns in metabolite and FA profiles between experimental groups, with variable importance in projection (VIP) scores used to identify key biomarkers. PLS-DA model performance was evaluated using the leave-one-out cross-validation (LOOCV) method, and model quality was reported in terms of prediction accuracy (%), *R*², and *Q*² (Cui et al., 2019). Metabolites and FA with VIP scores greater than 1.0 and *P*-values <0.05 were identified as potential discriminative biomarkers (Cui et al., 2019). The compounds with *P*-values between 0.10 to 0.05 were considered to demonstrate a trend toward significance. Additionally, *in silico* pathway enrichment analysis was conducted to explore altered metabolic and FA biosynthesis pathways, referencing the *Sus scrofa* pathway library in the Kyoto Encyclopedia of Genes and Genomes (KEGG) database.

## 3 Results

### 3.1 Sow and individual piglet and litter performances

The effects of maternal zinc supplementation on sow, individual piglet, and litter performances are presented in Table 2. TRT exhibited higher BFT on day 109 of gestation (*P* = 0.020) and day 21 of lactation (*P* = 0.023), as well as greater relative BFT loss during lactation (*P* = 0.024) than CON sows. The TB, BA, and SB did not differ, but MM was higher in TRT than in CON sows (*P* = 0.030). For individual piglets, birth weight and weight at 24 h after birth were unaffected. However, piglets nursed by TRT tended to have higher CI (*P* = 0.065) and were heavier on day 21 of age than those piglets nursed by CON sows (*P* = 0.116).

**Table 2:** Sow, individual piglet, and litter performances from sows fed a standard diet (CON), or sows fed a standard diet top dressed with 1 g/sow/day of bis-glycinate bound zinc during late gestation until day 21 of lactation (TRT) (Least-square means ± SEM).

Parameters	Group		
	CON	TRT	*P*-value
**Sow performance, *n***	18	18	
Parity^1^	2.7 ± 1.7	3.0 ± 2.0	
Backfat thickness, mm			
At day 85 of gestation	16.2 ± 1.2	17.8 ± 1.2	0.353
At day 109 of gestation	15.1 ± 1.2	19.4 ± 1.2	0.020
At day 21 of lactation	12.9 ± 1.0	16.4 ± 1.0	0.023
Relative backfat loss during lactation, %	11.8 ± 1.0	15.2 ± 1.0	0.024
Total number of piglets born/litter	15.1 ± 0.7	16.2 ± 0.7	0.324
Number of piglets born alive/litter	13.7 ± 0.6	13.4 ± 0.6	0.724
Stillborn piglets/litter, %	5.5 ± 1.4	7.2 ± 1.4	0.400
Mummified fetuses/litter, %	3.6 ± 1.6	8.6 ± 1.6	0.030
Number of functional teats, *n*	14.1 ± 0.2	14.1 ± 0.2	0.961
Colostrum yield, kg	4.30 ± 0.17	4.55 ± 0.17	0.302
Milk yield, kg/day			
Between day 3–10 of lactation	8.77 ± 0.29	8.62 ± 0.28	0.708
Between day 10–21 of lactation	9.76 ± 0.44	9.46 ± 0.43	0.629
**Individual piglet performance, *n***	246	241	
Piglet weight at birth, kg	1.42 ± 0.05	1.44 ± 0.05	0.785
Piglet weight at 24 h after birth, kg	1.51 ± 0.06	1.56 ± 0.06	0.556
Colostrum intake, g	330.1 ± 12.5	362.8 ± 12.5	0.065
**Litter performance, *n***	18	18	
On day 3 of age			
Average piglet weight, kg	1.76 ± 0.07	1.89 ± 0.08	0.263
Litter size^1^	13.0 ± 1.6	11.7 ± 2.2	
On day 10 of age			
Average piglet weight, kg	3.09 ± 0.14	3.19 ± 0.15	0.603
Litter size^1^	12.1 ± 1.6	11.6 ± 1.9	
On day 21 of age			
Average piglet weight, kg	4.83 ± 0.21	5.32± 0.22	0.116
Litter size^1^	12.0 ± 1.9	11.1 ± 2.1	

1 Means ± SD was reported instead of the Least Squares of the means ± SEM.

### 3.2 Incidence of diarrhea in piglets

**Fig. 1:**
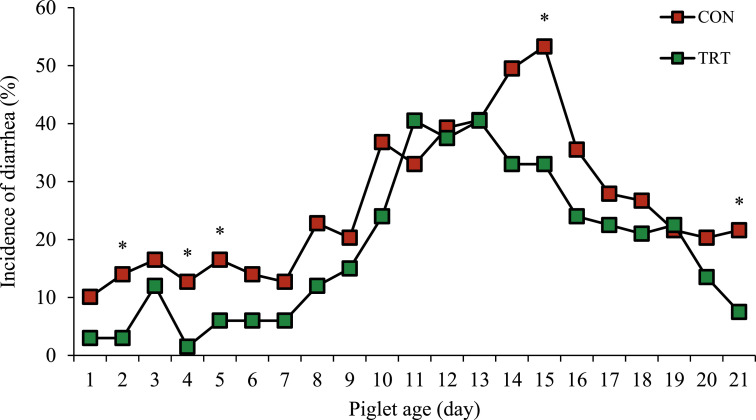
The incidence of diarrhea from piglets nursed by sows fed a standard diet (CON; red color) or piglets nursed by sows fed a standard diet top dressed with 1 g/sow/day of bis-glycinate bound zinc during late gestation until day 21 of lactation (TRT; green color). * Indicates a significant difference at *P* < 0.05.

The incidence of diarrhea was illustrated in Fig. 1, with a lower incidence observed in piglets nursed by TRT than those nursed by CON sows, particularly on days 2, 4, 5, 15, and 21 of age (*P* < 0.05; For all).

### 3.3 Concentration of serum MDA, colostrum Ig, and colostrum and milk major composition

The effects of maternal zinc supplementation on serum MDA, colostrum Ig, and colostrum and milk major composition are shown in Table 3. TRT exhibited higher serum MDA concentrations than CON sows (*P* = 0.033). No differences were observed in colostrum IgG, IgA, or IgM concentrations, nor in the major composition of colostrum and transient milk between groups. However, TRT exhibited higher contents of fat (*P* = 0.023) and total solids (*P* = 0.014), while lower lactose (*P* = 0.028) in mature milk than CON sows.

**Table 3:** Serum MDA concentrations, colostrum Ig concentrations, and major composition of colostrum and milk from sows fed a standard diet (CON), or sows fed a standard diet top dressed with 1 g/sow/day of bis-glycinate bound zinc during late gestation until day 21 of lactation (TRT) (Least-squares means ± SEM).

Parameters	Group		
	CON	TRT	*P*-value
**Number of samples, *n***	18	18	
**Serum, collected within 1h after the onset of farrowing**			
MDA, μmol/mL	5.0 ± 0.8	7.3 ± 0.7	0.033
**Colostrum, collected within 1h after the onset of farrowing**			
Immunoglobulin G, mg/mL	42.2 ± 4.4	45.2 ± 4.1	0.626
Immunoglobulin A, mg/mL	9.7 ± 1.3	8.5 ± 1.2	0.523
Immunoglobulin M, mg/mL	4.6 ± 0.5	4.6 ± 0.5	0.999
Protein, g/100 *g*	14.4 ± 0.5	15.6 ± 0.5	0.104
Casein, g/100 *g*	11.5 ± 0.4	12.4 ± 0.4	0.136
Fat, g/100 *g*	6.2 ± 0.6	6.2 ± 0.6	0.975
Lactose, g/100 *g*	2.7 ± 0.1	2.7 ± 0.1	0.965
Total solid, g/100 *g*	23.8 ± 0.6	25.1 ± 0.6	0.111
**Transient milk, collected on day 3 of lactation**			
Protein, g/100 *g*	5.5 ± 0.3	5.7 ± 0.3	0.632
Casein, g/100 *g*	4.4 ± 0.2	4.3 ± 0.2	0.772
Fat, g/100 *g*	9.7 ± 0.9	11.3 ± 1.0	0.243
Lactose, g/100 *g*	4.5 ± 0.1	4.3 ± 0.2	0.304
Total solid, g/100 *g*	21.3 ± 1.	22.7 ± 1.1	0.360
**Mature milk, collected on day 10 of lactation**			
Protein, g/100 *g*	4.2 ± 0.1	4.4 ± 0.1	0.292
Casein, g/100 *g*	3.8 ± 0.1	3.7 ± 0.1	0.457
Fat, g/100 *g*	7.0 ± 0.3	8.0 ± 0.3	0.023
Lactose, g/100 *g*	4.9 ± 0.1	4.6 ± 0.1	0.028
Total solid, g/100 *g*	18.1 ± 0.3	19.1 ± 0.73	0.014

### 3.4 Non-volatile polar metabolite profiles of sow colostrum and milk

**Fig. 2:**
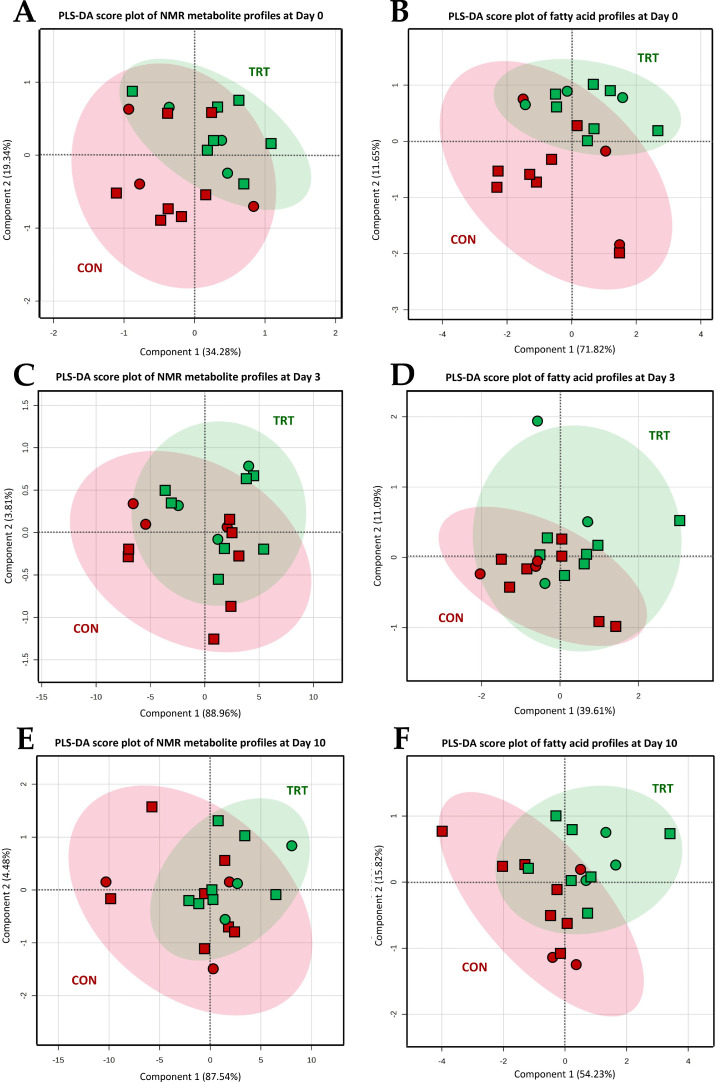
PLS-DA score plots for the comparison of non-volatile polar metabolite (panel A – C) and fatty acid profiles (panel D – F) of colostrum (panel A & D), transient milk (panel B & E) and mature milk (panel C & F) samples collected from sows fed a standard diet (Red color; *n* = 10), or sows fed a standard diet top dressed with 1 g/sow/day of bis-glycinate bound zinc during late gestation until day 21 of lactation (Green color; *n* = 10). Mammary secretion samples from primiparous (⬤) and multiparous (⬛) sows are differently symbolized.

**Fig. 3:**
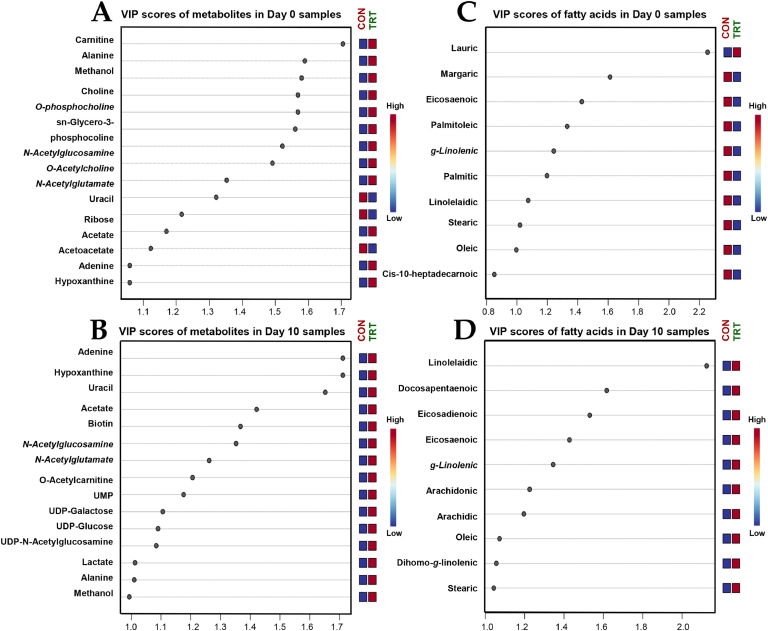
PLS-DA-derived VIP scores accountable for the discrimination of non-volatile polar metabolite (panel A & B) and fatty acid profiles (panel C & D) of colostrum (Panel A & C) and mature milk (Panel B & D) samples collected from sows fed a standard diet (CON; *n* = 10), or sows fed a standard diet top dressed with 1 g/sow/day of bis-glycinate bound zinc during late gestation until day 21 of lactation (TRT; *n* = 10).

A non-targeted ^1^H-NMR metabolomics approach enabled the identification of 38 non-volatile polar metabolites, including alcohols, amines, amino acids and derivatives, carbohydrates and derivatives, organic acids, and lipid derivatives, in sow colostrum and milk. For more details, see Supplementary materials (**Table S1**). PLS-DA score plots were generated separately for colostrum, transient milk, and mature milk, as demonstrated in Fig. 2. The results revealed a distinction of metabolite profiles between CON and TRT in colostrum (Fig. 2A, prediction accuracy = 73.59 %, *R*² = 0.538, and *Q*² = 0.432) and maturate milk (Fig. 2C, prediction accuracy = 59.13 %, *R*² = 0.562, and *Q*² = 0.457), whereas transient milk samples did not show a marked distinction between groups (Fig. 2B). The potential biomarker metabolites accountable for the discrimination with VIP scores greater than 1.0 in colostrum (Fig. 3A) and mature milk (Fig. 3B) were statistically evaluated and summarized in **Table S1**. The results demonstrated a distinct metabolic differentiation emerging as lactation progressed. In mature milk, significant differences were observed between groups for *O*-acetylcarnitine (*P* = 0.049), *O*-acetylcholine (*P* = 0.049), *sn*‑glycero-3-phosphocoline (*P* = 0.041), creatine (*P* = 0.029), uridine monophosphate (UMP) (*P* = 0.024), citrate (*P* = 0.028), and dimethylamine (*P* = 0.039) concentrations. In addition to this, certain metabolites, including carnitine, choline, *O*-phosphocholine, adenine, creatinine, hypoxanthine, *N*-acetylglutamate, and biotin, showed a borderline significant trend (0.05 < *P* < 0.06).

### 3.5 Fatty acid profiles of sow colostrum and milk

The GC/MS-FAME analysis revealed a total of 23 FA in sow colostrum, transient milk, and mature milk in this study. For more details, see Supplementary materials (**Table S2**). Consistent with the comparative metabolite profiling, three separate PLS-DA models were performed for the comparison of FA profiles among samples collected on the same day (Fig. 2). The results corresponded well with the metabolite pattern recognition, showing a distinction of metabolite profiles between groups in colostrum (Fig. 2D, prediction accuracy = 75.27 %, *R*² = 0.653, and *Q*² = 0.357) and mature milk (Fig. 2F, prediction accuracy = 85.34 %, *R*² = 0.546, and *Q*² = 0.428). In contrast, no clear distinction between the two groups was detected in transient milk FA profiles (Fig. 2E). In addition, the FA recognized as key discriminators based on VIP scores higher than 1.0 in colostrum (Fig. 3C) and mature milk (Fig. 3D) were subjected to statistical analysis and are presented in **Table S2**. It was found that the concentrations of arachidic acid (C20:0) (*P* = 0.021), linolelaidic acid (C18:2n6) (*P* = 0.007), eicosadienoic acid (C20:2n6) (*P* = 0.025), and arachidonic acid (C20:4n6) (*P* = 0.027) in mature milk differed significantly between CON and TRT groups. Indeed, variation in the contents of hexanoic acid (C6:0), stearic acid (C18:0), oleic acid (C18:1n9), and docosapentaenoic acid (C22:5n3) exhibited a strong trend towards significant difference (0.05 < *P* < 0.07).

### 3.6 Metabolic pathway analysis of altered metabolites

**Fig. 4:**
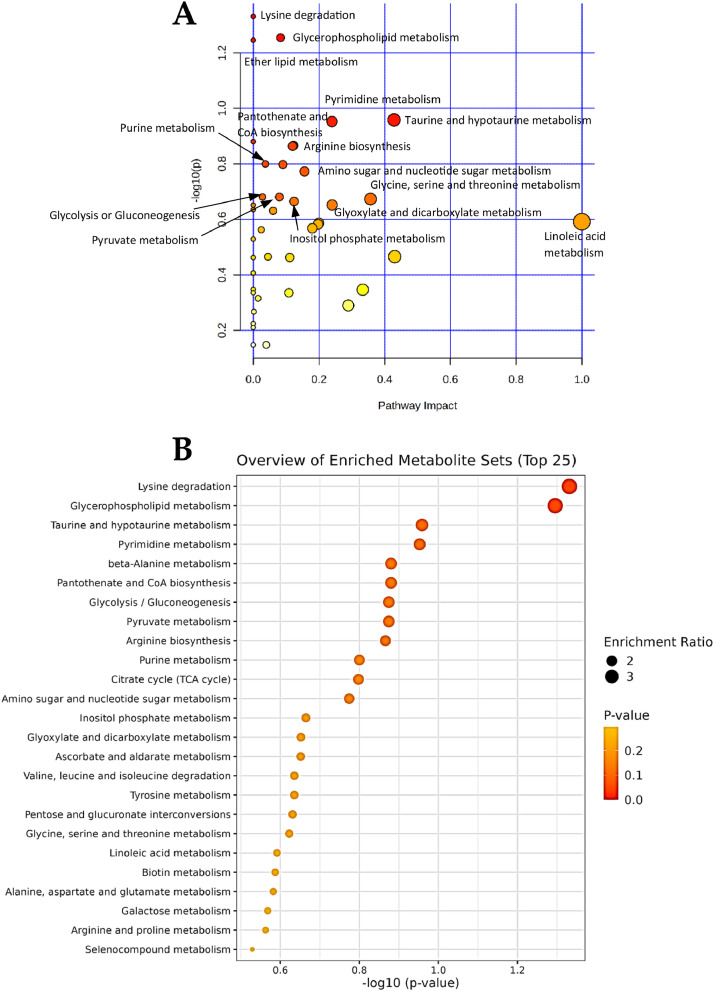
Alterations in the metabolic pathways in colostrum and mature milk between sows (*Sus scrofa*) fed a standard diet, or sows fed a standard diet top dressed with 1 g/sow/day of bis-glycinate bound zinc during late gestation until day 21 of lactation. (A) scatter plots of combined results from pathway impact and pathway topology analyses. The x-axis indicates the pathway impact, and y-axis indicates the pathway enrichment. Bubbles with darker color and larger size represent higher *P*-value from enrichment analysis and greater impact from the pathway topology analysis, respectively. (B) metabolite sets enrichment overview. The size of circles indicates the enrichment ratio, while the color represents the *P*-value, therefore, on the x-axis, higher value represents more significant associations between the metabolite set and the pathway.

To identify the most relevant metabolic pathways altered by maternal zinc supplementation, the ^1^H-NMR-derived metabolomic and GC-derived lipidomic data of colostrum and mature milk were combined and subjected to the *in silico* pathway enrichment analysis (Fig. 4). A higher *P*-value and impact value in Fig. 4A reflect the more relevant metabolic pathways in *Sus scrofa,* which could be differentially regulated in CON and TRT. The results revealed that lysine degradation, glycerophospholipid metabolism, ether lipid metabolism, taurine and hypotaurine metabolism, pyrimidine metabolism, pantothenate and CoA biosynthesis, arginine biosynthesis, glycolysis, and pyruvate metabolism were the top 10 substantial pathways perturbed. Furthermore, the 25 major pathways that can potentially be affected in sows between groups are presented in Fig. 4B

**Fig. 5:**
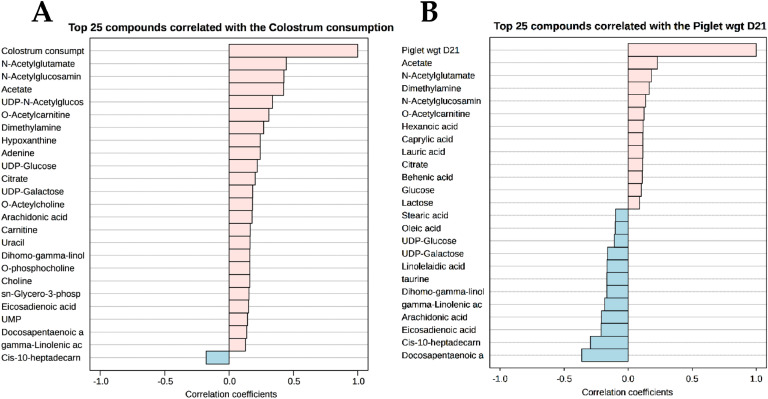
Correlation coefficient plots of the top non-volatile polar metabolites and fatty acids, represented as horizontal bars, with colors in light pink indicating positive correlations and light blue indicating negative correlations, to the colostrum consumption of piglets (A) and the average piglet weight on day 21 of age (B).

Pearson’s correlation analysis was conducted to identify metabolites and FA associated with improved piglet performance (Fig. 5). The results revealed that increased CI by piglets could be positively correlated with the concentrations of *N*-acetylglutamate, *N*-acetylglucosamine, acetate, UDP-*N*-acetylglucosamine, *O*-acetylcarnitine dimethylamine, adenine, hypoxanthine, UDP-glucose, and citrate in sow colostrum and milk (Fig. 5A). Furthermore, the higher average body weight of piglets on day 21 could be positively associated with the levels of acetate, *N*-acetylglutamate, dimethylamine, *N*-acetylglucosamine, *O*-acetylcarnitine, hexanoic acid, caprylic acid (C8:0), lauric acid (C12:0), citrate, behenic acid (C22:0), glucose and lactose in sow colostrum and milk (Fig. 5B).

## 4 Discussion

Zinc is an essential trace mineral that regulates growth and supports various metabolic functions in animals (Alimohamady et al., 2019; Duffy et al., 2023), including roles in nutrient utilization and absorption (He et al., 2019). In this study, zinc-supplemented sows exhibited higher BFT on day 109 of gestation compared with non-supplemented sows. Although BFT on day 85 of gestation was numerically higher in zinc-supplemented sows, no difference between groups was observed at this earlier stage, suggesting that the effect became more evident over time. This finding is consistent with previous studies showing that zinc supplementation above adequate levels enhances gastrointestinal enzyme activity, promotes nutrient digestion, and improves body condition (Holen et al., 2020; Jing et al., 2009). In addition, studies in weanling piglets have reported higher average daily gain and feed intake following zinc supplementation, potentially mediated by improved appetite regulation and palatability (Barszcz et al., 2019; Brandão-Neto et al., 1995). Collectively, these findings support a role of bis-glycinate-bound zinc supplementation in enhancing nutrient utilization in sows, contributing to the higher BFT observed on day 109 of gestation. However, individual feed intake was not measured, and initial BFT at the onset of supplementation may have influenced subsequent changes in body condition. Therefore, further studies controlling for baseline BFT and monitoring individual feed intake are required to confirm this effect.

Besides the benefits of zinc supplementation on nutrient utilization, a higher incidence of MM was observed in zinc-supplemented sows in this study. Fetal mummification is a multifactorial condition influenced by parity, litter size, uterine capacity, environmental factors, and infectious diseases (Borges et al., 2005; Dial et al., 1992; van der Lende & van Rens, 2003). It can occur from approximately day 35 of gestation until term, with higher occurrence during early fetal development, shortly after mid-gestation, and around day 100 of gestation, corresponding to critical phases of placental development (van der Lende & van Rens, 2003). Previous studies have shown that a lower placental production index is associated with an increased risk of fetal mummification, likely reflecting limited uterine capacity to sustain fetal growth during periods of rapid fetal development (Borges et al., 2005; Lefebvre, 2015; Wu et al., 1998). In addition, higher maternal BFT has been associated with a lipotoxic placental environment characterized by reduced angiogenesis and impaired nutrient transport (Liang et al., 2018; Saben et al., 2014; Zhou et al., 2018), which compromises placental efficiency and increases susceptibility to fetal loss. Therefore, the higher incidence of MM in zinc-supplemented sows coincides with higher BFT during late gestation, suggesting an indirect association mediated through altered placental function rather than a direct effect of zinc supplementation. However, placental function was not directly evaluated, and this interpretation requires further investigation.

Colostrum and milk serve as the primary nutrient source for neonatal piglets, providing essential components such as fat, protein, casein, lactose, Ig, metabolites, and FA (Hurley et al., 2015; Settachaimongkon et al., 2023). Although CY was not significantly different, a numerical increase was observed in zinc-supplemented sows, consistent with the tendency for higher CI observed in piglets nursed by zinc-supplemented sows. This pattern suggests a perinatal benefit of maternal zinc supplementation. Although birth weight did not differ between groups, intrinsic traits that influence CI, such as time to stand and inter-birth interval, may underlie the observed pattern (Laothong et al., 2024; Nuntapaitoon et al., 2019b). Consistent with this, Hammers et al. (2024) showed that feeding elevated maternal zinc during gestation improves farrowing kinetics and piglet resilience to farrowing stress, which could contribute to the greater CI.

On day 21 of age, piglets nursed by zinc-supplemented sows exhibited greater body weight than those nursed by non-supplemented sows. However, the difference in litter size between groups (11.1± 2.1 piglets/litter in zinc-supplemented sows vs. 12.0 ± 1.9 piglets/litter in non-supplemented sows) may have influenced this finding, as larger litter size increases the competition for milk, potentially reducing piglet growth (Kobek-Kjeldager et al., 2020; Lee et al., 2024). Importantly, the number of functional teats exceeded the number of piglets throughout lactation and did not differ between groups. In addition, estimated MY during days 3–10 and 10–21 of lactation was comparable between groups (8–10 kg/day), which is within the normal range for sows raised under tropical conditions (Khamtawee et al., 2021; Ruampatana et al., 2024, 2025b; 2025c). These findings indicate that milk production capacity did not account for the observed difference in piglet body weight. Instead, the higher body weight is more likely associated with differences in colostrum and milk nutritional composition. Nevertheless, the lack of litter size standardization remains a limitation and should be controlled in future studies.

Nutritional composition of colostrum and milk is a key determinant of piglet growth and survival. In this study, maternal zinc supplementation did not affect the major nutritional composition of colostrum or transient milk. However, in mature milk, zinc-supplemented sows exhibited lower lactose content and higher fat and total solids compared with non-supplemented sows. Lactose serves as a primary energy source during lactation and is closely associated with milk production (Buranakarl et al., 2021; Zhao et al., 2021), whereas milk fat and associated biomolecular components provide additional energy, support growth, and contribute to antimicrobial defense (Hansen et al., 2012; Settachaimongkon et al., 2023). The reduction in lactose is likely related to zinc-mediated interference with manganese uptake in mammary epithelial cells (Liu et al., 2021; Permyakov et al., 2000), which impairs lactose synthesis. In contrast, the higher milk fat content reflects enhanced mobilization of maternal fat reserves during lactation, which also accounts for the greater total solids observed in mature milk. As milk fat content is associated with depletion of BFT or loin muscle (Costermans et al., 2020), this finding aligns with the greater BFT loss observed in zinc-supplemented sows. In dairy cows, increased fat mobilization is associated with elevated plasma MDA levels, a marker of lipid peroxidation and oxidative stress (Abuelo et al., 2014; Rodríguez et al., 2020; Sordillo & Aitken, 2009), which is consistent with the higher serum MDA observed at farrowing in zinc-supplemented sows. Collectively, these findings indicate that bis-glycinate-bound zinc supplementation modulates maternal fat mobilization, thereby altering milk composition.

In addition to the observed effects on piglet growth and milk composition, piglets nursed by zinc-supplemented sows exhibited a lower incidence of diarrhea. Maternal zinc supplementation has been reported to support intestinal health in piglets by modulating gut microbiota, including suppression of opportunistic pathogens such as *Campylobacteriaceae* and *Escherichia* (Oh et al., 2021) and by downregulating the expression of tumor necrosis factor-alpha, thereby improving intestinal permeability (Diao et al., 2021). During lactation, zinc transfer to piglets occurs primarily through milk and is largely derived from maternal reserves, with secretion levels exceeding placental transfer during gestation (Davin et al., 2015; King, 2002), indicating the importance of maternal zinc status in neonatal supply. However, milk zinc concentration was not measured in this study, which limits direct interpretation of zinc-mediated effects. Therefore, metabolomic and lipidomic analyses were applied to characterize broader changes in milk biomolecular composition associated with maternal zinc supplementation, rather than to attribute the observed effects to zinc-specific mechanisms alone.

Non-targeted ^1^H-NMR metabolomics analysis demonstrated that maternal zinc supplementation altered the non-volatile metabolite profile of sow colostrum and milk. PLS-DA indicated a lactation-stage–dependent effect, as group separation was limited in transient milk but increased from colostrum to mature milk, suggesting a cumulative dietary influence. Colostrum or milk metabolites are mostly derived from blood or are synthesized by mammary epithelial cells (Rezaei et al., 2016). Therefore, the observed shifts may be attributed to zinc-mediated modulation of systemic metabolism or mammary epithelial cells’ biosynthetic activity. In mature milk, zinc-supplemented sows exhibited higher concentrations of *O*-acetylcarnitine, *O*-acetylcholine, *sn*‑glycero-3-phosphocholine, creatine, UMP, citrate, and dimethylamine. These metabolites are involved in energy metabolism, methylation reactions, nucleotide biosynthesis, and membrane integrity (Curtasu et al., 2016; Luise et al., 2020; Picone et al., 2018; Settachaimongkon et al., 2023). In particular, higher levels of *O*-acetylcarnitine, creatine, and citrate are consistent with enhanced mitochondrial β-oxidation and tricarboxylic acid cycle activity (Luise et al., 2020; Picone et al., 2018), which may increase the energetic value of milk and support piglet growth. Similarly, higher concentrations of choline derivatives (*O*-acetylcholine and *sn*‑glycero-3-phosphocholine) may facilitate membrane fluidity, neural, and intestinal development (Curtasu et al., 2016; Luise et al., 2020), potentially contributing to improved piglet performance. Other metabolites with borderline significance (hypoxanthine, *N*-acetylglutamate, and biotin) may also be biologically relevant despite limited statistical power. These findings suggested that maternal zinc supplementation optimizes colostrum and milk metabolites, improving piglet growth.

Consistent with ^1^H-NMR metabolomic profiling, lipidomic analysis demonstrated that maternal zinc supplementation altered the FA profile of sow colostrum and mature milk, with clear group separation in these lactation stages. Colostrum and milk FA are generally influenced by parity, lactation stage, diet, and body fat reserve (Amdi et al., 2013; Holen et al., 2023; Settachaimongkon et al., 2023). As parity, basal diet, and sampling time points were comparable between groups, the observed differences in milk FA profiles likely reflect zinc-associated changes in maternal lipid mobilization and mammary lipid metabolism. In mature milk, zinc-supplemented sows exhibited higher concentrations of arachidic, linolelaidic, eicosadienoic, and arachidonic acids, FA known to support neonatal energy supply, metabolism, and immune development (Settachaimongkon et al., 2023). Arachidic acid contributes to sphingolipid and ceramide synthesis in piglets (Wang et al., 2023), and its higher abundance may indicate enhanced FA elongation under improved maternal metabolic status with zinc supplementation. Linolelaidic acid may originate from endogenous lipid oxidation or isomerization, suggesting modifications in oxidative lipid metabolism consistent with the elevated serum MDA in zinc-supplemented sows (Kapusta et al., 2018). Eicosadienoic and arachidonic acids participate in membrane phospholipid remodeling and act as eicosanoid precursors essential for gut, immune, and neural development in neonates (Hahn et al., 2020; Huang et al., 2011). In addition, trends toward increased medium- and long-chain FA (hexanoic acid, stearic acid, and docosapentaenoic acid) further suggest enhanced lipid mobilization and mammary biosynthesis under zinc supplementation (Zhang et al., 2018). Together with the milk metabolite shifts and the observed increases in piglet body weight and reduced diarrhea incidence, these coordinated alterations in milk metabolite and FA composition provide mechanistic support for the association between maternal zinc supplementation and improved piglet production performance.

Integrated pathway enrichment analysis further substantiated the biological relevance of the observed metabolomic and lipidomic alterations in sow colostrum and mature milk after supplementation with zinc. The results demonstrated that the most perturbed pathways included lysine degradation, glycerophospholipid and ether lipid metabolism, and pyrimidine metabolism, reflecting coordinated shifts in amino acid catabolism, membrane lipid remodeling, and nucleotide metabolism. These pathways are vital for maintaining cellular structure, supporting tissue growth, and modulating biosynthetic activity in the sow mammary gland (Zhang et al., 2018). Additionally, the enrichment of taurine and hypotaurine metabolism, pantothenate and CoA biosynthesis, glycolysis, and pyruvate metabolism suggests enhanced antioxidant capacity and energy metabolism in lactating sows (Gormley et al., 2024). These findings imply that zinc supplementation not only alters milk biomolecular composition but may also improve milk functional properties by modulating pathways associated with oxidative-stress mitigation, cellular energetics, and immunomodulatory potential (Blavi et al., 2021; Duffy et al., 2023). Consistent with this interpretation, piglets nursed by zinc-supplemented sows demonstrated a lower diarrhea incidence and greater CI and weights on day 21 of age. Moreover, several milk metabolites and FA, specifically *N*-acetylglutamate, dimethylamine, acetate, citrate, and hexanoic acid, were positively correlated with enhanced CI and greater weight on day 21 of age in piglets. Together, these findings underscore the physiological relevance of zinc-induced changes in milk composition as contributors to improved piglet production performance.

## 5 Conclusions

Maternal supplementation with bis-glycinate bound zinc in sows during late gestation and lactation altered milk composition and improved piglet performance. Zinc-supplemented sows exhibited greater BFT loss during lactation, coinciding with higher fat and lower lactose levels in mature milk. Metabolomic and lipidomic analyses further revealed an enrichment of various milk metabolites and FA, which corresponded with reduced pre-weaning diarrhea and a tendency toward higher piglet body weight. From a production standpoint, improved piglet health and early growth suggest potential economic benefits through improved pre-weaning performance and reduced health-related challenges. These findings support maternal bis-glycinate bound zinc supplementation as a practical nutritional strategy to enhance milk quality and support neonatal health in swine production.

## Declaration of generative AI use

During the preparation of this manuscript, the authors used ChatGPT-Plus (https://chatgpt.com/) to check the linguistic aspect. After using this tool, the authors reviewed and edited the content as needed and took full responsibility for the content of the publication.

## Ethical statement

The animal procedures used in this study were conducted in accordance with the ethical standards for the care and use of animals in research. All experimental protocols were reviewed and approved by the Institutional Animal Care and Use Committee (IACUC) of Chulalongkorn University, Thailand (Approval number: 2331017). Every effort was made to minimize animal suffering and to use the minimum number of animals necessary to achieve scientific objectives.

## CRediT authorship contribution statement

**Jakavat Ruampatana:** Writing – review & editing, Writing – original draft, Methodology, Investigation, Data curation, Conceptualization. **Sarn Settachaimongkon:** Writing – review & editing, Writing – original draft, Methodology, Investigation, Data curation, Conceptualization. **Nathathai Soodsaward:** Validation, Investigation, Data curation. **Banthita Chuaydamrong:** Validation, Investigation, Data curation. **Ponlatorn Rukklang:** Validation, Investigation, Data curation. **Suphacha Boonyasantisuk:** Validation, Investigation, Data curation. **Suphasorn Ratchatakajornkit:** Validation, Investigation, Data curation. **Unchean Yamsrikaew:** Validation, Investigation, Data curation. **Kunaporn Homyog:** Methodology, Formal analysis. **Rawiwan Kuabphimai:** Methodology, Formal analysis. **Lampetch Vimuktalop:** Methodology, Formal analysis. **Junpen Suwimonteerabutr:** Methodology, Formal analysis. **Morakot Nuntapaitoon:** Writing – review & editing, Validation, Supervision, Project administration, Methodology, Investigation, Funding acquisition, Formal analysis, Data curation.

## Declaration of competing interest

The authors declare that they have no known competing financial interests or personal relationships that could have appeared to influence the work reported in this paper.

## Appendix Supplementary materials

Image, application 1
