# Supplementary material for: Maternal bis-glycinate bound zinc supplementation alters sow performance and milk metabolomic-lipidomic profiles and mitigates piglet diarrhea
Source: Vet Anim Sci. 2026 May 12;33:100697. doi: 10.1016/j.vas.2026.100697 (PMC13197804; doi:10.1016/j.vas.2026.100697)
Supplement: Supplementary file 1 [file mmc1.docx]

**Supplementary materials**

**Table S1** Concentration of colostrum and milk non-volatile metabolites from sows fed a standard diet (CON) or sows fed a standard diet top dressed with 1 g/sow/day of bis-glycinate bound zinc during late gestation until day 21 of lactation (TRT) by lactation stage (colostrum, transient milk, and mature milk).

| Metabolites^1^ | Colostrum | | SEM^2^ | *P*-value | Transient milk | | | SEM | *P*-value | | Mature milk | | | SEM | *P*-value |
| --- | --- | --- | --- | --- | --- | --- | --- | --- | --- | --- | --- | --- | --- | --- | --- |
|  | CON | TRT |  |  | CON | TRT | |  |  |  | CON | | TRT |  |  |
| Alcohols and polyols | | | | | | | | | | | | | | | |
| Methanol | 12.030 | 12.076 | 0.032 | 0.334 | 11.510 | 11.693 | | 0.057 | 0.036 | | 11.547 | | 11.737 | 0.062 | 0.044 |
| Myo-Inositol | 13.345 | 13.337 | 0.017 | 0.746 | 13.298 | 13.370 | | 0.037 | 0.183 | | 13.450 | | 13.569 | 0.045 | 0.076 |
| Amines | | | | | | | | | | | | | | | |
| Carnitine | 13.043 | 13.092 | 0.027 | 0.223 | 12.895 | 12.987 | | 0.056 | 0.257 | | 12.828 | 12.982 | | 0.052 | 0.053 |
| Choline | 13.078 | 13.123 | 0.026 | 0.240 | 12.933 | 13.023 | | 0.056 | 0.276 | | 12.855 | 13.011 | | 0.053 | 0.052 |
| O-Acetylcarnitine | 12.737 | 12.766 | 0.016 | 0.209 | 12.319 | 12.443 | | 0.079 | 0.281 | | 12.262 | 12.493 | | 0.077 | 0.049 |
| O-Acteylcholine | 13.233 | 13.276 | 0.021 | 0.166 | 12.994 | 13.095 | | 0.059 | 0.242 | | 12.942 | 13.112 | | 0.057 | 0.049 |
| O-phosphocholine | 13.078 | 13.123 | 0.026 | 0.240 | 12.933 | 13.023 | | 0.056 | 0.276 | | 12.855 | 13.011 | | 0.053 | 0.052 |
| sn-Glycero-3-phosphocoline | 13.269 | 13.313 | 0.022 | 0.168 | 13.035 | 13.145 | | 0.057 | 0.190 | | 12.987 | 13.158 | | 0.055 | 0.041 |
| Amino acids and derivatives | | | | | | | | | | | | | | | |
| Adenine | 12.148 | 12.178 | 0.018 | 0.259 | 11.489 | 11.696 | | 0.092 | 0.129 | | 11.418 | 11.746 | | 0.111 | 0.052 |
| Alanine | 12.706 | 12.751 | 0.018 | 0.083 | 11.972 | 12.137 | | 0.091 | 0.215 | | 12.023 | 12.217 | | 0.075 | 0.862 |
| Betain | 13.442 | 13.460 | 0.020 | 0.512 | 13.376 | 13.455 | | 0.043 | 0.207 | | 13.473 | 13.598 | | 0.047 | 0.074 |
| Creatine | 13.064 | 13.093 | 0.015 | 0.183 | 13.073 | 13.178 | | 0.030 | 0.024 | | 13.256 | 13.401 | | 0.043 | 0.029 |
| Creatinine | 12.869 | 12.881 | 0.016 | 0.583 | 12.533 | 12.631 | | 0.052 | 0.199 | | 12.525 | 12.675 | | 0.052 | 0.054 |
| Creatine phosphate | 13.469 | 13.456 | 0.020 | 0.651 | 13.555 | 13.618 | | 0.032 | 0.186 | | 13.738 | 13.854 | | 0.043 | 0.075 |
| Glutamate | 13.563 | 13.553 | 0.019 | 0.728 | 13.630 | 13.700 | | 0.034 | 0.164 | | 13.813 | 13.929 | | 0.044 | 0.081 |
| Glycine | 13.292 | 13.263 | 0.020 | 0.331 | 13.382 | 13.442 | | 0.033 | 0.223 | | 13.566 | 13.676 | | 0.044 | 0.091 |
| Hypoxanthine | 12.148 | 12.178 | 0.018 | 0.259 | 11.489 | 11.700 | | 0.092 | 0.129 | | 11.418 | 11.746 | | 0.111 | 0.052 |
| N-Acetylglutamate | 13.070 | 13.109 | 0.018 | 0.149 | 12.658 | 12.812 | | 0.082 | 0.198 | | 12.612 | 12.854 | | 0.085 | 0.059 |
| Threonine | 12.412 | 12.439 | 0.018 | 0.308 | 11.705 | 11.867 | | 0.088 | 0.211 | | 11.759 | 11.936 | | 0.071 | 0.092 |
| Taurine | 12.228 | 12.231 | 0.029 | 0.943 | 11.919 | 12.043 | | 0.049 | 0.091 | | 12.074 | 12.211 | | 0.048 | 0.060 |
| UMP | 12.851 | 12.841 | 0.018 | 0.692 | 12.668 | 12.807 | | 0.051 | 0.071 | | 12.507 | 12.732 | | 0.064 | 0.024 |
| Uracil | 11.947 | 11.984 | 0.055 | 0.630 | 11.437 | 11.599 | | 0.111 | 0.317 | | 11.479 | 11.795 | | 0.134 | 0.111 |
| Uridine | 13.364 | 13.364 | 0.017 | 0.987 | 13.306 | 13.377 | | 0.042 | 0.241 | | 13.453 | 13.572 | | 0.047 | 0.089 |
| Carbohydrates and derivatives | | | | | | | | | | | | | | | |
| Glucose | 13.058 | 13.047 | 0.023 | 0.749 | 13.334 | | 13.260 | 0.028 | | 0.078 | 13.580 | 13.463 | | 0.042 | 0.064 |
| Lactose | 14.493 | 14.477 | 0.020 | 0.563 | 14.598 | | 13.536 | 0.035 | | 0.225 | 14.829 | 14.715 | | 0.044 | 0.087 |
| N-Acetylglucosamine | 13.217 | 13.261 | 0.018 | 0.102 | 12.762 | | 12.924 | 0.091 | | 0.220 | 12.664 | 12.923 | | 0.093 | 0.065 |
| Ribose | 11.549 | 11.514 | 0.040 | 0.545 | 10.960 | | 11.075 | 0.092 | | 0.387 | 11.232 | 13.353 | | 0.101 | 0.406 |
| UDP-Galactose | 12.227 | 12.223 | 0.032 | 0.937 | 11.972 | | 12.101 | 0.080 | | 0.268 | 11.821 | 12.033 | | 0.081 | 0.082 |
| UDP-Glucose | 12.317 | 12.302 | 0.027 | 0.694 | 12.016 | | 12.133 | 0.071 | | 0.262 | 11.900 | 12.109 | | 0.083 | 0.091 |
| UDP-N-Acetylglucosamine | 12.922 | 12.924 | 0.018 | 0.961 | 12.637 | | 12.753 | 0.062 | | 0.204 | 12.513 | 12.720 | | 0.076 | 0.068 |
| Organic acids | | | | | | | | | | | | | | | |
| Acetate | 12.651 | 12.684 | 0.017 | 0.190 | 12.152 | | 12.316 | 0.099 | | 0.260 | 12.120 | 12.392 | | 0.102 | 0.076 |
| Acetoacetate | 11.988 | 11.955 | 0.029 | 0.442 | 11.794 | | 11.872 | 0.040 | | 0.188 | 11.949 | 12.082 | | 0.048 | 0.064 |
| Biotin | 12.417 | 12.442 | 0.016 | 0.296 | 11.862 | | 12.043 | 0.088 | | 0.166 | 11.640 | 11.091 | | 0.089 | 0.051 |
| Citrate | 13.051 | 13.071 | 0.019 | 0.459 | 12.650 | | 12.812 | 0.063 | | 0.088 | 12.612 | 12.797 | | 0.055 | 0.028 |
| Dimethylamine | 12.457 | 12.465 | 0.020 | 0.780 | 12.039 | | 12.192 | 0.068 | | 0.131 | 11.999 | 12.186 | | 0.059 | 0.039 |
| Glycolate | 13.500 | 13.490 | 0.020 | 0.750 | 13.583 | | 13.647 | 0.033 | | 0.184 | 13.765 | 13.882 | | 0.044 | 0.075 |
| Lactate | 12.602 | 12.630 | 0.018 | 0.282 | 11.910 | | 12.073 | 0.093 | | 0.229 | 11.937 | 12.131 | | 0.078 | 0.096 |
| Vitamin |  |  |  |  |  | |  |  | |  |  |  | |  |  |
| Ascorbate | 13.140 | 13.141 | 0.017 | 0.971 | 13.180 | | 13.265 | 0.035 | | 0.099 | 13.365 | 13.487 | | 0.046 | 0.075 |

^1^ Data are presented as the Least square mean and SEM, both calculated from log_10_ transformed peak area values [arbitrary unit].

^2^ Greatest standard error of the mean (SEM).

**Table S2** Concentration of colostrum and milk fatty acids from sows fed a standard diet (CON) or sows fed a standard diet top dressed with 1 g/sow/day of bis-glycinate bound zinc during late gestation until day 21 of lactation (TRT) by lactation stage (colostrum, transient milk, and mature milk).

| Fatty acid^1^ | Colostrum | | SEM^2^ | *P*-value | Transient milk | | SEM | *P*-value | Mature milk | | | SEM | *P*-value |
| --- | --- | --- | --- | --- | --- | --- | --- | --- | --- | --- | --- | --- | --- |
|  | CON | TRT |  |  | CON | TRT |  |  | CON | TRT | |  |  |
| Saturated fatty acids (SFA) | | | | | | | | | | | | | |
| Hexanoic acid |  |  |  |  |  |  |  |  | 5.348 | 5.431 | | 0.030 | 0.066 |
| Caprylic acid |  |  |  |  |  |  |  |  | 5.601 | 5.584 | | 0.040 | 0.763 |
| Capric acid | 5.224 | 5.160 | 0.04 | 0.552 | 6.045 | 6.108 | 0.054 | 0.425 | 6.441 | 6.414 | | 0.044 | 0.669 |
| Lauric acid | 5.620 | 0.568 | 0.070 | 0.154 | 6.451 | 6.449 | 0.034 | 0.970 | 6.570 | 6.590 | | 0.039 | 0.710 |
| Myristic acid | 7.183 | 7.162 | 0.056 | 0.777 | 7.667 | 7.684 | 0.028 | 0.674 | 7.686 | 7.711 | | 0.038 | 0.638 |
| Pentadecanoic acid | 6.334 | 6.300 | 0.045 | 0.598 | 6.645 | 6.624 | 0.031 | 0.650 | 6.620 | 6.597 | | 0.038 | 0.673 |
| Palmitic acid | 8.488 | 8.410 | 0.048 | 0.263 | 8.742 | 8.728 | 0.020 | 0.634 | 8.647 | 8.686 | | 0.029 | 0.354 |
| Margaric acid | 6.574 | 6.468 | 0.048 | 0.135 | 6.839 | 6.780 | 0.036 | 0.270 | 6.469 | 6.501 | | 0.041 | 0.580 |
| Stearic acid | 7.902 | 7.835 | 0.055 | 0.404 | 8.178 | 8.134 | 0.038 | 0.425 | 7.837 | 7.938 | | 0.037 | 0.067 |
| Arachidic acid | 6.515 | 6.522 | 0.06 | 0.934 | 6.814 | 6.767 | 0.030 | 0.279 | 6.510 | 6.626 | | 0.032 | 0.021 |
| Behenic acid |  |  |  |  | 6.108 | 6.082 | 0.037 | 0.629 | 5.853 | 5.938 | | 0.032 | 0.085 |
| Monounsaturated fatty acids (MUFA) | | | | | | | | | | | | | |
| Palmitoleic acid | 7.256 | 7.169 | 0.059 | 0.307 | 8.028 | 8.035 | 0.029 | 0.866 | 8.032 | | 8.044 | 0.037 | 0.818 |
| Cis-10-heptadecarnoic acid | 6.712 | 6.656 | 0.05 | 0.438 | 6.883 | 6.830 | 0.046 | 0.431 | 6.459 | | 6.489 | 0.055 | 0.709 |
| Oleic acid | 8.716 | 8.651 | 0.047 | 0.342 | 8.989 | 9.947 | 0.034 | 0.390 | 8.675 | | 8.780 | 0.038 | 0.068 |
| Eicosaenoic acid | 6.628 | 6.535 | 0.086 | 0.452 | 7.071 | 6.986 | 0.051 | 0.255 | 6.595 | | 6.734 | 0.054 | 0.088 |
| Omega-3 polyunsaturated fatty acids (PUFA) | | | | | | | | | | | | | |
| Linolenic acid | 7.236 | 7.242 | 0.057 | 0.932 | 7.475 | 7.514 | 0.037 | 0.467 | 7.413 | | 7.498 | 0.040 | 0.158 |
| Docosapentaenoic acid | 6.494 | 6.511 | 0.058 | 0.831 | 6.554 | 6.562 | 0.044 | 0.900 | 6.137 | | 6.294 | 0.056 | 0.062 |
| Omega-6 polyunsaturated fatty acids (PUFA) | | | | | | | | | | | | | |
| Linolelaidic acid | 6.441 | 6.371 | 0.061 | 0.425 | 6.739 | 6.658 | 0.050 | 0.263 | 6.229 | | 6.435 | 0.048 | 0.007 |
| Linoleic acid | 8.543 | 8.508 | 0.050 | 0.635 | 8.698 | 8.700 | 0.027 | 0.965 | 8.509 | | 8.592 | 0.032 | 0.084 |
| gamma-Linoleic acid | 6.657 | 6.576 | 0.076 | 0.460 | 7.026 | 6.972 | 0.036 | 0.313 | 6.234 | | 6.364 | 0.060 | 0.140 |
| Eicosadienoic acid | 6.834 | 6.820 | 0.053 | 0.850 | 7.081 | 7.064 | 0.044 | 0.781 | 6.597 | | 6.746 | 0.043 | 0.025 |
| Dihomo-gamma-linolenic | 6.573 | 6.534 | 0.053 | 0.608 | 6.697 | 6.606 | 0.053 | 0.026 | 6.132 | | 6.235 | 0.053 | 0.185 |
| Arachidonic acid | 7.166 | 7.126 | 0.055 | 0.610 | 7.264 | 7.120 | 0.097 | 0.308 | 6.857 | | 6.976 | 0.035 | 0.027 |

^1^ Data are presented as the Least square mean and SEM, both calculated from log_10_ transformed peak area values [arbitrary unit].

^2^ Greatest standard error of the mean (SEM).
